# The silent storm: How climate, food commodity prices, and political stability fuel Somalia’s inflation crisis

**DOI:** 10.1371/journal.pone.0356334

**Published:** 2026-11-04

**Authors:** Abdimalik Ali Warsame, Khadar Ahmed Dirie, Amir Mohamud Mohamed

**Affiliations:** 1 Department of Economics, Faculty of Economics and Management Sciences, Hormuud University, Mogadishu, Somalia; 2 Department of Economics, Faculty of Managerial Sciences, Abdullah Gül University, Kayseri, Türkiye; Lusofona University of Humanities and Technologies: Universidade Lusofona de Humanidades e Tecnologias, PORTUGAL

## Abstract

In fragile economies, inflation is a critical macroeconomic issue for household well-being in vulnerable nations. As prices increase, at-risk families are forced to diminish essential consumption, remove children from formal education, and forsake long-term developmental goals to satisfy immediate survival requirements. In this regard, this study examines the impact of climate change, food commodity prices, and political stability on inflation in Somalia. Unlike previous studies, this study employs a novel Kernel regularized least squares (KRLS) machine learning method, time-varying Granger causality, and an innovative supremum right-tail Augmented Dickey-Fuller test to examine explosive tendencies in data series. The results of the explosive-behavior analysis identify one period of statistically explosive dynamics in the consumer price index (2006–2022), food commodity prices (2010–2014), and sugar commodity prices (2010–2012). In addition, the time-varying Granger causality between the sampled variables is observed. The empirical results of the KRLS method indicated that food commodity prices and average temperature have a positive and increasing marginal effect on inflation, whereas average rainfall and political stability reduce it with a decreasing marginal effect. These results are also robust for various econometric methods and disaggregate food commodity prices, including sugar, oil, and meat commodities. The study findings highlight the importance of implementing a composite strategy that enhances climate resilience, stabilizes food systems, and improves political governance as a possible solution to fighting inflation in fragile economies such as Somalia.

## 1 Introduction

Inflation is not merely an economic indicator; it is a persistent force that erodes the purchasing power of vulnerable families worldwide. When prices rise, families skip meals, children miss school, and dreams vanish. Globally, food inflation rose by 23.5% between 2020–2023, forcing 71 million people into poverty [1,2]. This rise in global inflation, caused by conflicts, climate change, and economic problems, has resulted in a sharp increase in poverty and food insecurity among people in low- and middle-income countries. Low-income families are especially threatened by inflation because they spend a significant share of their income on necessities [3]. Many developing countries have inflation-related food shortages, and it is mostly the poor who are significantly affected. The unpredictable changes in food prices cause poverty and hunger in these families, mainly through the rise in general prices [4]. Therefore, inflation usually makes it harder to get enough food, increases poverty, and reduces access to basic services such as healthcare and education [5]. Largely because of inflationary problems and falling salaries caused by the world’s economic and food crises, poverty and hunger have increased in most low-income countries [6]. Since inflation raises the costs of most goods and services, it is expected that its effects on well-being in poor regions will take a long time and will persist.

Empirical evidence demonstrates that climate change – changing rainfall patterns and increasing temperatures – leads to complicated and lasting increases in prices, especially among mainly agricultural societies. This global pattern also has important regional implications. Recently, Kotz et al. [7] examined data from over 27,000 monthly consumer price indices worldwide and found that food inflation rises by about 0.17% with every increase of 1°C in average temperature. Even though climate change affects every region in the world, its effect is more pronounced in the least developed countries, where there is a lack of climate adaptation and mitigation strategies. Climate change is an extremely serious and pressing concern in Somalia, as the country is considered one of the most climate-vulnerable countries in the world [8,9]. The effect of climatic consequences is prevalent in the country since agriculture is the main source of livelihoods and income. Due to their dependence on agriculture, such communities are not very resilient to economic changes, and weather-related disasters can ruin their protective systems. For example, Somalia frequently experiences floods that reduce agricultural productivity, which makes the soil too wet, leads to soil erosion, and encourages deforestation [10,11]. Environmental issues hurt crops and break up supply chains, which results in supply-driven rises in prices and makes it harder for people to afford staple foods. As time passes, the effects of these shocks increase, which makes it tougher for the economy to recover.

Furthermore, since Somalia depends so much on imports for its staple foods, its economy is vulnerable to both global disturbances and changes in global markets. Global commodity price shocks also affect inflation. This vulnerability was evident during the 2022 Russia-Ukraine conflict since access to the Black Sea was disrupted and prices for grain and fuel surged worldwide [12,13]. When commodities make up more than 35% of the overall cost of living in a country like Somalia, adverse global events immediately cause inflation to rise at home. Even after international markets stabilize, prices of goods that dominate people’s daily expenses remain quite high. Persistently high prices are generally the outcome of structural factors within the economy. External scarcity and household problems, such as bottlenecks, fuel prices, lack of market controls, and hoarding, are shown by the rising food inflation in Somalia. Consequently, 2024’s inflation remains pronounced, as the World Bank points out, despite deflation that has hit the global markets for commodities [14]. In addition, the country’s poor road network and fragmented distribution system do not allow easy movement of food to regions in need, as price arbitrage is hard [15]. As a result, small logistical errors can worsen the effect of supply shortages and make food prices uneven in different regions, all of which strengthen the country’s food inflation. Global dependency is only one reason for Somalia’s inflationary risks; the main threat also comes from the ongoing domestic issues that do not allow the economy to react properly or remain stable.

Another significant aspect of inflation control is political stability. Political instability is likely to raise inflation and make it more volatile, especially in developing nations and high-inflation economies. A nation’s struggles with political instability often cause food prices to rise as economic policies change frequently [16]. Due to constant instability in places such as Somalia, there are ongoing challenges with keeping organizations running, controlling economic activities, and responding well to changes in supply. This situation leads to higher inflation, mainly when it comes to food, since governance gaps allow food prices to change, investment in infrastructure decreases, and crisis planning is blocked [17]. Salma et al. [18] showed that political instability makes inflation more unstable by lowering public trust in institutions and stopping consistent policies. The situation in Somalia is especially challenging since prolonged climate adversities, a set of divided federal states, and weak governance have created a weak base for managing the country’s economy [19]. Somalia’s central bank finds it difficult to function independently since it has limited authority over monetary matters, and currency movements are made through informal and unregulated channels [20,21]. A lack of centralized management hinders using effective anti-inflation policies and leads to fluctuating currency values. During 2024, Somalia’s total fiscal deficit went up to 8.9% of GDP because the government was forced to spend more on helping people affected by droughts and floods [14]. When public institutions are weak, attempts to make up for the budget gap may rely on help from external donors and only produce temporary results. The absence of proper governance encourages people to pursue rents. In countries where the government is weak, armed groups seize the opportunities of taxing supply lines during natural disasters. Such transaction costs increase the final consumer prices, disproportionately burdening poor households [17].

Yet nowhere is the inflation crisis more acute than in Somalia, where the Global Hunger Index [22] lists the country as the world’s hungriest, and almost 51.3% of its population is undernourished [23]. It demonstrates that developing countries are more at risk when they face several shocks at the same time. The Somali National Bureau of Statistics [24] revealed that 54% of the country’s people are below the national poverty line, and most of them depend on rainfed agriculture [25,26]. There is a combination of serious environmental problems, unsteady prices for commodities, and political issues that make this country’s situation complex [27]. From 2022 to 2023, Somalia was hit by extreme drought, and moments later, devastating floods caused 3.8 million people to flee and entirely disrupted local food production [27,28]. Due to these environmental disasters, the country currently faces even more difficulty from inflation, which was 5.5% in 2024. Consequently, many people are unemployed, and their income and purchasing power decrease during inflation crises. Sustained climate and economic crises have caused families to lose their assets, which in turn leads to more poverty and danger. The 2022 Integrated Household Budget Survey discloses that poverty is more common among those who are nomadic and those who live in cities, which highlights existing gaps in the country’s socio-economic system [14].

Although inflation is a critical determinant of household welfare and macroeconomic stability, little empirical work has been carried out in Somalia to explain the determinants and temporal dynamics of inflation. More broadly, previous studies have covered climate change [29], disruptions in commodity markets [13], or political factors [18] separately; these combined influences have not often been explored, particularly in the Somali context. Therefore, there is limited empirical evidence on how their interrelated relationships influence inflation in a fragile institutional context like Somalia. This gap is especially important because Somalia is highly vulnerable to extreme weather events, relies significantly on imported food commodities, and has experienced prolonged political instability, which may have a combined effect on the dynamics of domestic prices. To address this gap, instead of a single determinant, the current study presents an integrated climate, commodity-price, political-stability, and macroeconomic analysis of Somalia’s inflation. The analysis employs complementary econometric methods to examine various aspects of these relationships. In particular, the Kernel-Based Regularized Least Squares (KRLS) technique is used to explore potentially nonlinear and heterogeneous associations without assuming a fixed functional form; the ARDL framework is used to determine long-run and short-run relationships; the supremum right-tailed Augmented Dickey–Fuller (ADF) approach is used to detect periods of statistically explosive dynamics, and time-varying Granger analysis is applied to test the hypothesis that the predictive relationship between the selected variables and inflation is time-varying. Moreover, the contribution of the study goes beyond adding extra econometric methods; it rests in its substantive emphasis on the dynamics of inflation in Somalia as well as its holistic treatment of environmental, commodity-market, and political factors. This focus also differentiates the current study from the authors’ Somalia-related study published earlier. While Warsame et al. [8] explored the relationship among climate change, conflict, and food security with time-varying Granger analysis, the current study investigates how inflation is related to climate conditions, global food commodity prices, and political stability with inflation as the outcome variable. The two studies share a common context in Somalia and some methodological aspects, but vary in research question, dependent variable, empirical relationship, and policy implications.

The remaining sections of the paper are organised in the following manner: The second section provides a concise overview of the existing theoretical and empirical literature related to the study. The third section presents the data used and the methods employed in the study. The fourth section presents the findings of the empirical analysis and provides a discussion of these results. The final section concludes the study and offers relevant policy implications.

## 2 Literature review

### 2.1 Theoretical literature review

Inflation is a multifaceted macroeconomic phenomenon. This study is an interdisciplinary one that examines how political factors, global commodity prices, and climate change drive inflation in a fragile country, Somalia. The study applies several theories of inflation, such as demand-pull inflation, cost-push inflation, and agricultural supply shocks. Political stability, global food commodity prices, and climate change influence inflation via proven theoretical mechanisms. Political instability undermines the credibility of monetary and fiscal policies, increases risk premiums, devalues the exchange rate, and disrupts production, thereby inducing both cost-push and demand-pull inflation [30,31]. Global food commodity prices affect domestic inflation through cost-push dynamics and imported inflation, since increased international food costs are transmitted to domestic markets via global price pass-through and fluctuations in exchange rates [32,33]. Climate change exacerbates inflation chiefly via agricultural supply shocks and energy-related disturbances—extreme weather events diminish crop yields, elevate food prices, and escalate production and transportation expenses, thereby contributing to consumer price inflation [34,35]. These dynamics interrelate, as climate-induced agricultural failures elevate global food costs, while political instability exacerbates exchange-rate pass-through, collectively heightening inflationary pressures.

### 2.2 Empirical literature review

The nexus among climate change, food commodity prices, and political stability has become a fundamental challenge to attaining macroeconomic stability in developing fragile states [36,37]. This review examines these dimensions individually—climate change, food commodity prices, and political stability—and their respective and aggregate effects on inflation in the context of fragile states, based on insights from empirical literature and evidence across the globe.

To begin with, the issue of climate change has taken a central role in economic policymaking and growing evidence shows that climate change may have an effect on macroeconomic variables, mainly inflation [29,38–41]. For instance, Qi et al. [29] established that climate risk, quantified using the German watch index, causes an increase in short-term inflation, particularly in low-income countries, through reductions in central bank interest rates and increases in food prices. The study by Li et al. [39], which establishes a significant, positive correlation between temperature fluctuations and inflation across countries highlights the importance of increasing energy demand and uncovers a nonlinear association moderated by GDP per capita. Odongo et al. [38], considering Eastern and Southern Africa, demonstrated that food price rises are caused by rainfall variability and food inflation (importation), and oil prices and subsidies influence the overall inflation. Emediegwu [40] evaluated the asymmetric impacts of El Niño Southern Oscillation (ENSO) on food prices in Sub-Saharan Africa and pointed out that the effects vary significantly across the regions, and particularly, in maize-reliant regions. A similar result has been reported by Erdogan et al. [41] in Nigeria. Overall, the reviewed studies indicate that climate-induced inflation is region- and income-sensitive, and it requires specific economic, environmental, and monetary policies to be reduced. Nevertheless, these studies focus on climate-related causes of inflation and do not take into account the potential impact of food commodity prices and political stability at the same time. This also leaves the question of whether inflationary pressures arising from the climate remain when these other major drivers are included together – especially in climate-sensitive and fragile economies.

Climate change is not the only factor determining inflation. There is also evidence in the literature that food commodity price volatility is a crucial element in the development of inflationary pressures, especially in economies where food accounts for a substantial proportion of household consumption and the consumer price index. De Gregorio [4] stressed the inflationary effects of commodity price shocks, particularly food. The study found that food inflation has a greater second-round impact on core inflation compared to energy prices and so should be closely monitored by monetary policymakers. Using the Indonesian case, Fadhilah and Ayu [42] revealed that changes in main food commodities, particularly rice, red chili, chicken, and eggs, greatly influence inflation in both the short and long term in Pematang Siantar City, with red chili presenting the strongest influence. Likewise, Irawati et al. [43] demonstrated that the prices of rice, red chilies, and onions have a large effect on inflation volatility in North Sumatra Province, whereas garlic did not make a significant contribution, illustrating the commodity-based inflation sensitivity. Radukici et al. (2015) discovered that the primary cause of inflation is food price volatility in Serbia, which is induced by seasonal and natural factors of supply. Lastly, Davidson et al. [44] reported that global food commodity prices and oil prices have a considerable effect on domestic food price inflation in the UK, and the longevity of price shocks is equally important as their size. Overall, the existing studies confirm that food commodity price shocks are an overwhelming and multifaceted inflationary agent that necessitates concerted policy actions that are dependent upon the organization of domestic food markets. However, the evidence mainly addresses food commodity prices as a standalone source of inflationary pressure. Analyzing the food-price effects alone might thus be limited in explaining the inflation dynamics in economies where agricultural production is highly climate-dependent, and market volatility and disruption also occur in the political arena.

Furthermore, another important determinant of inflation is political stability. Political instability is likely to raise the level of inflation and the volatility of inflation rates, especially in developing countries and high-inflation economies. Barugahara [45] found that political instability, as captured by the state failure and fragility indices, positively and significantly boosts inflation volatility in 49 African countries. In a similar vein, Ghanayem et al. [46] concentrated on the MENA region and broke down the concept of political instability into five divergent aspects through factor analysis. Their results are inconclusive but informative. Regime instability (which amplifies inflation volatility) is countered by government instability (e.g., frequent cabinet changes) that can decrease it due to transitory stabilization policies or externally imposed control. Such dynamics are specified with several GARCH-type specifications, which provide robustness. Khan and Saqib [47] concluded that political instability is a robust driver of inflation especially via nonmonetary mechanisms whereby uncertainty erodes investor confidence together with economic predictability in Pakistan. A larger cross-country view is presented by Telatar et al. [48], who found that political instability is a major cause of inflation in high-inflation and developing nations. Institutional quality, they stress, is central to the question of how political factors are transformed into economic ones, with democratic institutions and political liberties moderating this association. Generally, most studies confirm that political stability is not only a vital condition to prevent inflation but also to limit its uncertainty, particularly in weak and developing countries. This highlights the necessity of institutional change and democratic stabilization to provide macroeconomic robustness. However, the political stability-inflation literature has tended to focus on the impacts of shocks to politics and inflation without considering the links to climate and food commodity prices. It is therefore unclear how political stability is related to inflation in the presence of both environmental and commodity-market pressures, particularly in the fragile economy of Somalia.

Overall, the literature examined indicates a strong body of knowledge about the impact of climate conditions, food commodity prices, and political stability on inflation; however, there are significant gaps. First, these determinants have largely been examined separately, providing limited evidence on their joint relationship with inflation. Second, there has been relatively little research on the possibility that these relationships may change over time. Third, empirical research on these interconnections is limited in fragile and climate-sensitive economies like Somalia. A comprehensive framework is necessary to tackle these gaps by simultaneously addressing the environmental, commodity-market, political, and macroeconomic aspects of inflation, and by permitting nonlinear and time-varying relationships. Therefore, this study investigates the interlinkage of Somalia’s climate variables, food commodity prices, political stability, and inflation by applying complementary KRLS and time-varying Granger causality approaches, as well as ARDL analysis to examine long-run and short-run relationships. Finally, the study brings Somalia-specific evidence on the multi-faceted and dynamic nature of inflation and offers a more holistic foundation for policy discussion in this context.

## 3 Data and methods

### 3.1 Data

Somalia is an agrarian and import-dependent country, which is vulnerable to both global commodity-price and climate shocks. Additionally, the country has been encountering political unrest since 1991. The weakened government institutions that previously controlled inflation have lost their effectiveness. This study examines the determinants of inflation in Somalia, which is a crucial macroeconomic issue in the country. This study adopts an interdisciplinary approach to examine the determinants of inflation by ascertaining the impact of climate change—average rainfall and temperature, food commodity prices, and political stability on the consumer price index in Somalia. The study utilizes annual time series data spanning from 1996 to 2022. The selection of the sampling period for the data is based on its availability. Moreover, to obtain robust results, we also incorporated disaggregated food commodity prices, such as sugar, oil, cereal, and meat, into the model. Detailed explanations of the data sources and descriptions are displayed in Table 1.

**Table 1:** Data sources and descriptions.

Variable	Code	Descriptions	Sources
Inflation	CPI	Consumer Price Index (CPI) gauges the average temporal variation in the prices of a “market basket” of goods and services, including food, housing, and transportation, commonly bought by households.	IMF & Somali National Bureau of Statistics
Rainfall	AR	Average annual precipitation (mm)	World Governance Indicators,World Bank
Political stability	PS	Political Stability and Absence of Violence/Terrorism: Estimate	World Governance Indicators,World Bank
Temperature	AT	Average annual temperature, (°C)	SESRIC,OIC database
Food commodity prices	FPI	Global food price index	Food & Agriculture Organization (FAO)
Sugar food commodity	Sugar	Global sugar price index	Food & Agriculture Organization (FAO)
Oil commodity prices	Oil	Global oil price index	Food & Agriculture Organization (FAO)
Cereal commodity prices	Cereal	Global cereal price index	Food & Agriculture Organization (FAO)
Meat commodity prices	Meat	Global meat price index	Food & Agriculture Organization (FAO)
Real gross domestic product	GDP	Gross domestic product (Constant, 2015)	SESRIC,OIC database

### 3.2 Methods

#### 3.2.1 Kernel-based regularized least squares.

To investigate the relationship between inflation and the selected independent variables, this study primarily employed the Kernel-based Regularized Least Squares (KRLS) approach. It is a machine learning technique specifically developed to address the limitations of traditional regression and classification models that rely on linear or additive assumptions [49]. KRLS offers an interpretive framework similar to that of generalized linear models, particularly in terms of deriving the marginal effects of explanatory variables. It surpasses conventional linear regression methods, which are often prone to misspecification bias. Firstly, KRLS delivers more precise results due to its flexible modeling of the conditional expectation function and the mean derivatives of parameters, enabling the detection of varying effects across regressors. Secondly, it effectively tackles the issue of overfitting by incorporating an optimal regularization function that penalizes model complexity. Thirdly, KRLS is capable of estimating complex models that include interaction terms, nonlinear relationships, and non-additive structures with high efficiency and accuracy [49]. KRLS approximates an unknown response surface within a reproducing kernel Hilbert space (RKHS), allowing nonlinearities and interactions to emerge from the data without imposing a predetermined functional form. The regularization component controls model complexity and reduces the risk of overfitting.

The KRLS approach is based on the assumption that the observations are independent and identically distributed (i.i.d.). Each observation is denoted by i, where $y_{i}$represents the dependent variable and $X_{i}$denotes the vector of explanatory variables corresponding to the i-th observation. To measure the similarity between observations according to their explanatory characteristics, KRLS uses a symmetric, positive semi-definite Gaussian kernel function, specified as:


$$\begin{array}{c} K(x_{i},x_{j})=\text{exp}\left(-\frac{∥x_{i}-x_{j}{∥}^{\text{2}}}{\text{2}\sigma^{\text{2}}}\right) \end{array}$$ (1)

$e^{x}\;$denotes the exponential function. While the term $∥x_{i}-x_{j}∥$represents the Euclidean distance between the covariate vectors of observations $x_{i}$and $x_{j}$. A key property of the KRLS approach is that the Gaussian kernel function reaches its maximum value when the two observations are identical ($x_{i}=x_{j}$) and gradually approaches zero as the distance between them increases. Consequently, $K(x_{i},x_{j})$serves as a measure of the similarity between the explanatory variable vectors of the two observations.

In this expression, e signifies the exponential function, while the term $∥x_{i}-x_{j}∥\;$measures the Euclidean distance between the covariate vectors of observations iand j. The Gaussian kernel reaches its maximum value when the two observations are identical ($X_{i}=X_{j}$) and progressively decreases toward zero as the distance between their covariate vectors surges. Therefore, the kernel function provides a quantitative measure of the similarity between the explanatory-variable vectors of the two observations.

From a similarity-based perspective, the target function fis approximated by a function drawn from a class of functions constructed according to the similarities among the observations, expressed as:


$$\begin{array}{c} f(x)=\sum_{i=\text{1}}^{N}c_{i}K(x,x_{i}), \end{array}$$ (2)

In Equation (2), K shows the kernel function, which evaluates the degree of similarity between the point of interest xand the covariate vector $X_{i}$, while $\alpha_{i}$ signifies the corresponding weight for each covariate vector.

The following regularized objective function is minimized by the KRLS estimator.


$$\begin{array}{c} \underset{f\in H}{\text{min}}\left\{\sum_{i=\text{1}}^{N}V(y_{i},f(x_{i}))+\lambda R(f)\right\}, \end{array}$$ (3)

where $V(y_{i},f(x_{i}))$ represents the loss function employed to quantify the prediction error related to each observation, R(f) shows the regularization term that penalizes model complexity, λ∈R is the regularization parameter that determines the trade-off between goodness-of-fit and model complexity, and H signifies the hypothesis space comprising all allowable functions.

For example, $X(x_{\text{1}},\ldots ,x_{d},\ldots ,x_{D})$ symbolize a dataset containing Nobservations and D-dimensional explanatory variables. Built on Equation (3), the observation-specific (pointwise) marginal effect of the d-th explanatory variable for the j-th observation is computed as


$$\begin{array}{c} \frac{\partial {\hat{y}}_{j}}{\partial x_{j}^{d}}=-\frac{\text{2}}{\sigma^{\text{2}}}\sum_{i=\text{1}}^{N}c_{i}\text{\textasciitilde{}exp}\left(-\frac{∥x_{i}-x_{j}{∥}^{\text{2}}}{\text{2}\sigma^{\text{2}}}\right)(x_{i}^{d}-x_{j}^{d}). \end{array}$$ (4)

Here ${\hat{y}}_{j}$ represents the predicted value of the dependent variable for observation j, $x_{j}^{d}$ signifies the d-th explanatory variable for observation j, $c_{i}$represents the kernel weights assessed from the data, and σ displays the kernel bandwidth parameter. Equation (4) reflects the marginal contribution of each explanatory variable separately across individual observations, thereby permitting the effects to vary across the sample.

The Average Marginal Effect (AME) is obtained by taking the mean of the observation-specific marginal effects across the entire sample:


$$\begin{array}{c} AME=-\frac{\text{2}}{N\sigma^{\text{2}}}\sum_{j=\text{1}}^{N}\sum_{i=\text{1}}^{N}c_{i}\text{exp}\left(-\frac{∥x_{i}-x_{j}{∥}^{\text{2}}}{\text{2}\sigma^{\text{2}}}\right)(x_{i}^{d}-x_{j}^{d}). \end{array}$$ (5)

Equations (4) and (5) enable KRLS to estimate marginal effects at the observation level without imposing a specific functional form. In contrast to conventional regression approaches, which typically assume constant marginal effects across observations, KRLS allows the estimated effects to differ across the sample. These observation-specific effects are summarized using the Average Marginal Effect (AME), along with the 25th percentile (P25), median (P50), and 75th percentile (P75), providing a clearer representation of the variation and heterogeneity in the estimated effects across observations.

The econometric model is specified as follows, referring to Odongo et al. [38], Li et al. [39], and Irawati et al. [43].


$$\begin{array}{c} \text{ln}{\text{CPI}}_{\text{t}}=\beta_{\text{0}}+\beta_{\text{1}}{\text{lnAR}}_{\text{t}}+{\beta_{\text{2}}\text{lnAT}}_{\text{t}}+\beta_{\text{3}}{\text{lnFPI}}_{\text{t}}+\beta_{\text{4}}{\text{lnPS}}_{\text{t}}{+\beta }_{\text{5}}{\text{lnGDP}}_{\text{t}}+\varepsilon_{\text{t}}\; \end{array}$$ (6)

where CPI, AR, AT, FPI, PS, and GDP stand for consumer price index, average rainfall, average temperature, global food commodity price index, political stability, and gross domestic product, respectively. $\beta_{\text{0}}$ is the intercept, $\beta_{\text{1}}$to $\beta_{\text{5}}$ are the coefficients of the explanatory variables. ln denotes logarithmic form and $\varepsilon_{\text{t}}$ for error term.

#### 3.2.2 Explosive behavior.

Political and economic shocks at both global and regional levels can trigger abnormal effects on production and consumption, resulting in what is termed “explosive behavior.” This refers to a typical pattern observed in environmental and macroeconomic variables over time. To investigate such dynamics, this study employs the innovative supremum right-tail Augmented Dickey-Fuller (ADF) unit root test. This method is based on the null hypothesis of a unit root; thus, rejecting the null suggests the presence of outliers, indicative of explosive behavior [50]. Due to the presence of explosive behavior in the data, we employed a time-varying Granger causality approach to better capture potential instabilities. Traditional VAR techniques are insufficient for estimating causality in the presence of regime shifts and structural breaks, which can lead to unstable relationships. In contrast, the time-varying Granger causality method addresses these limitations by accommodating such instabilities and providing robust statistical inference across different periods. Specifically, we implemented a reduced-form VAR model with a maximum of two lags and used a trimming parameter of 0.15, under the assumption of homoskedastic idiosyncratic shocks. We conducted four tests based on the null hypothesis of no Granger causality: the robust exponential Wald (ExpW), robust mean Wald (MeanW), optimal Nyblom (Nyblom), and quasi-likelihood ratio (QLR) tests [51].

#### 3.2.3 Time-varying granger causality framework.

This study also uses the time-varying Granger causality framework as a key component of its empirical strategy to assess whether the relationships between inflation and the selected explanatory variables change over time. In contrast to the standard Granger causality procedure, the time-varying approach permits the strength and significance of these relationships to differ across sample periods. This feature is particularly relevant in the presence of structural transformations and regime shifts, which can influence the stability of conventional Vector Autoregressive (VAR) models. Moreover, the approach enables the identification of particular periods during which the predictive relationships are statistically significant, thereby offering greater insight into their temporal evolution [52]. Three complementary procedures are considered within the time-varying Granger framework: the forward-expanding window approach, the rolling-window procedure, and the recursive-expanding window method developed by Phillips et al. [53]. All three procedures are implemented in this study using a corrective bootstrap algorithm.

The forward-expanding and rolling-window procedures rely on Wald statistics, whereas the recursive procedure is based on the supremum norm calculated from a sequence of Wald statistics obtained across the observations. Accordingly, the maximum values of the corresponding test statistics are defined as follows:

The forward-expanding window:


$$\begin{array}{c} M_{\text{1},t}^{b}=\begin{array}{c} Max\\ t\varepsilon [\tau_{\text{0}},\tau_{b}+\tau_{b}-\begin{array}{c} \text{1}] \end{array} \end{array}T_{\text{1},t}^{b} \end{array}$$ (7)

The rolling window:


$$\begin{array}{c} M_{t-\tau_{\text{0}}+\text{1},t}^{b}=\begin{array}{c} Max\\ t\varepsilon [\tau_{\text{0},}\tau_{b}-\begin{array}{c} \text{1}] \end{array} \end{array}T_{t-\tau_{\text{0}}+\begin{array}{c} \text{1} \end{array}}^{b} \end{array}$$ (8)

The recursive window:


$$\begin{array}{c} \overset{―}{{\text{M}}_{t}^{b}}\begin{array}{c} (\tau_{\text{0}} \end{array})=\begin{array}{c} Max\\ t\varepsilon [\tau_{\text{0},}\tau_{\text{0}}+\tau_{b}-\begin{array}{c} \text{1} \end{array} \end{array}\{T_{t}^{b}\left(\tau_{\text{0}}\right)\} \end{array}$$ (9)

#### 3.2.4 ARDL model.

To explore the relationship between inflation and the independent variables, this study further employed the ARDL bounds test. This dynamic econometric method is designed to examine long-run relationships within a time-series context. The ARDL approach offers several advantages over traditional cointegration tests. Specifically, it is particularly suitable for studies with small sample sizes and can effectively accommodate a combination of variables that are integrated of order zero, I (0), and order one, I (1). Moreover, the ARDL model is relatively straightforward to implement and interpret, as it relies on a single-equation framework. Another key benefit is its flexibility, allowing different lag lengths to be assigned to each variable in the model [54,55]. Our approach aims to explore potential long-run relationships among the variables under study by estimating an ARDL model as presented in Equation (10) below.


$$\begin{array}{cc} {\Delta \text{lnCPI}}_{\text{t}}= & \alpha_{\text{0}}+\Omega_{\text{1}}{\text{lnCPI}}_{\text{t}-\text{1}}+\Omega_{\text{1}}{\text{lnAR}}_{\text{t}-\text{1}}+\Omega_{\text{2}}{\text{lnAT}}_{\text{t}-\text{1}}+\Omega_{\text{3}}{\text{lnFPI}}_{\text{t}-\text{1}}+\Omega_{\text{4}}{\text{lnPS}}_{\text{t}-\text{1}}+\Omega_{\text{5}}{\text{lnGDP}}_{\text{t}-\text{1}}\\ & +\sum_{\text{i}=\text{1}}^{\text{p}}\varphi_{\text{1}}{\Delta \text{lnCPI}}_{\text{t}-\text{i}}+\sum_{\text{i}=\text{1}}^{\text{q}}\varphi_{\text{2}}{\Delta \text{lnAR}}_{\text{t}-\text{i}}+\sum_{\text{i}=\text{1}}^{\text{q}}\varphi_{\text{3}}{\Delta \text{lnAT}}_{\text{t}-\text{i}}+\sum_{\text{i}=\text{1}}^{\text{q}}{\varphi_{\text{4}}\Delta \text{lnFPI}}_{\text{t}-\text{i}}+\sum_{\text{i}=\text{1}}^{\text{q}}{\varphi_{\text{4}}\Delta \text{lnPS}}_{\text{t}-\text{i}}\\ & +\sum_{\text{i}=\text{1}}^{\text{q}}{\varphi_{\text{4}}\Delta \text{lnGDP}}_{\text{t}-\text{i}}+\;\epsilon_{\text{t}}\; \end{array}$$ (10)

In this equation, α₀ represents the constant term, Ω denotes the long-run coefficients, φ indicates the short-run coefficients, i refers to the lagged values, ∆ signifies the first differences, and p and q correspond to the optimal lag lengths for the respective variables.

## 4 Empirical results and discussion

### 4.1 Descriptive and correlation analysis

Table 2 reports the summary statistics of the sampled variables, such as mean, standard deviation, and maximum values. The examined variables have recorded mean values of 3.9 (inflation), 5.6 (average rainfall), 3.2 (average temperature), 4.4 (food commodity prices), −2.3 (political stability), and 22 (economic growth). Political stability has the lowest mean value (−2.3), whereas GDP has the highest mean value. Moreover, average temperature has the lowest standard deviation value (0.006) compared to other variables, which implies that its data dispersion is minimal. In contrast, the correlation results among the sampled variables are also reported in Table 2. It is observed that all the variables are positively correlated with inflation except political stability.

**Table 2:** Summary statistics.

Variable	Mean	Std. dev.	Min	Max		
lnCPI	3.962	.816	2.129	4.892		
lnAR	5.644	.096	5.456	5.853		
lnAT	3.295	.006	3.284	3.308		
lnFPI	4.455	.300	3.977	4.973		
PS	−2.347	.915	−3.312	0		
lnGDP	22.089	.591	21.185	22.955		
**Correlation**						
	**lnCPI**	**lnAR**	**lnAT**	**lnFPI**	**PS**	**lnGDP**
lnCPI	1.0000					
lnAR	0.3960	1.0000				
lnAT	0.4909	0.2444	1.0000			
lnFPI	0.8307	0.2810	0.2358	1.0000		
PS	−0.5522	0.0491	−0.3412	−0.5306	1.0000	
lnGDP	0.9337	0.4704	0.4985	0.7282	−0.3844	1.0000

### 4.2 Unit root test

Even though the KRLS method and time-varying Granger causality do not need pretesting of the variables, we reported the results of Augmented Dickey-Fuller (ADF) unit root test in Table 3. It revealed that our variables of interest are integrated at mixed orders, which is the combination of level I (0) and first difference I (1). Hence, we could proceed to the estimation of long-run cointegration since no variable is integrated of order two, I (2).

**Table 3:** Unit root tests (ADF).

Variable	At level	At first difference
lnCPI	−2.197	− 4.737***
lnFPI	−3.658**	−4.076
lnAR	−6.322***	−4.382**
lnAT	−5.108***	−7.921***
PS	−7.149***	−1.609
lnGDP	−3.269*	−2.936
lnSugar	−3.471	−4.804***
lnOil	−5.114***	−4.536***
lnCereal	−1.873	−4.194**
lnMeat	−3.163	−4.546***

*, **, and *** represent significance levels at 10%, 5%, and 1% respectively. The t-statistics reported are for the intercept and trend only.

### 4.3 Explosive behavior and time-varying Granger causality

The innovative supremum right-tail Augmented Dickey-Fuller unit root method is used to capture explosive behaviors [50,56]. The findings illustrated in Fig 1 indicate the rejection of the null hypothesis of a unit root within the right-tail 90–95% confidence interval. The rejection indicates the presence of anomalous values of explosive behavior among the variables. It reveals one explosive pattern in the consumer price index measured for inflation (2006–2022) and food commodity prices (FCP) (2010–2014). Additionally, an explosive trend is evident in sugar commodity prices (2010–2012), as illustrated in Fig 1(C). Following the identification of structural breaks in several key variables, the application of traditional Granger causality will yield unreliable results. To address this deficiency, we employed a novel and robust time-varying Granger causality method in the context of instabilities proposed by Rossi and Wang [51]. The usual VAR models and other causality methods do not work well for figuring out how variables affect each other because of instability from regime shifts and structural breaks. This strategy surpasses traditional causality from multiple angles. First, it examines the existence of instabilities and reaffirms the statistical significance of the causality test across different times within the sample. It also performs exceptionally well in evaluating the validity tests for reduced-form VAR models and VAR-based direct multistep (VAR-LP) predictive models.

**Fig 1:**
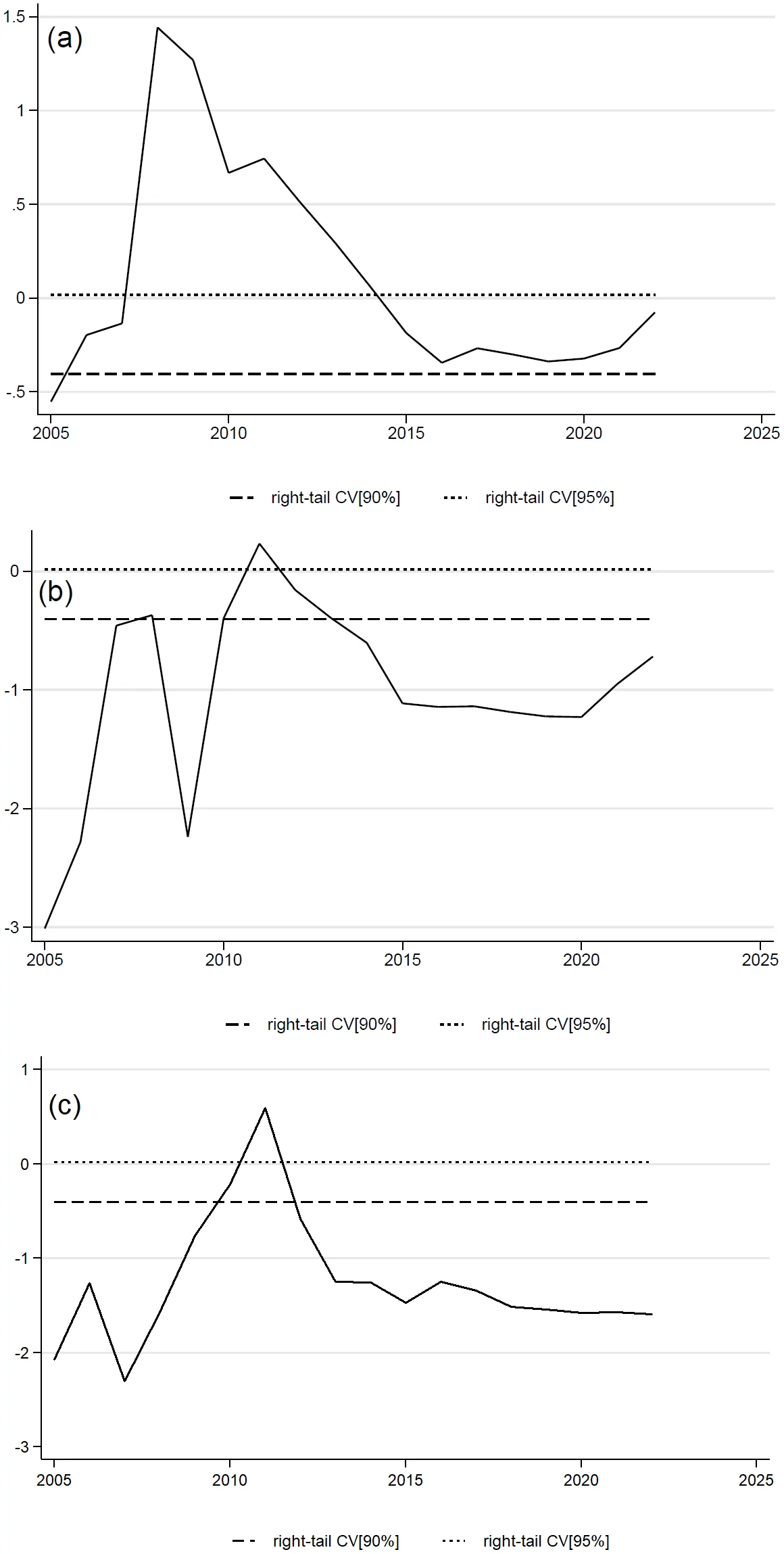
Date-stamping episodes for explosive behavior of (a) Consumer price index (b) Food commodity price, and (c) Sugar.

Time-varying Granger causality signifies the causal relationship between inflation and the relevant independent variables. To ascertain the significance of causation among the relevant factors, we utilized the 90th and 95th percentiles of the empirical distribution. Figs 2 and 3 present all the test statistics for the forward-expanding, recursive-evolving, and rolling-window methodologies. Two variables exert mutual causation when the statistical sequence surpasses the 90th and 95th percentiles. Fig 2 illustrates the causal relationship between food commodity prices and inflation. All three algorithms—forward, recursive, and rolling—exhibit distinct causality events and intervals of non-causality, with the exception of the rolling-window algorithm. In the forward result, two episodes of Granger causality (2004–2010 and 2012–2022) from food commodity prices to inflation are determined, with the peak period of the causality being in 2009. Similarly, there are two episodes of Granger causality from global commodity prices to inflation during 2006–2008 and 2008–2009, with the peak causality in 2009 in the recursive results. In the rolling window result, no significant causality from food commodity prices to inflation is observed. Notably, both the forward and recursive approaches produced similar results, underscoring that food commodity prices posed severe threats to food security in Somalia by increasing food prices in 2009 and 2011. Even though Somalia encountered severe droughts in 2011, global food price crises contributed to the drought developing into famine in Somalia [8]. Further, a similar trend of causality from sugar commodity prices to inflation is observed, as shown in Fig 3. Global sugar commodity price Granger-caused inflation in Somalia during 2004–2015, as shown by the forward result, whereas no significant causality is detected between them in the rolling window result. Finally, five episodes of causality (2005, 2009–2012, 2016–2017, 2018–2019, and 2021–2022) are observed from sugar commodity prices to inflation, with a peak causality period of 2016–2017.

**Fig 2:**
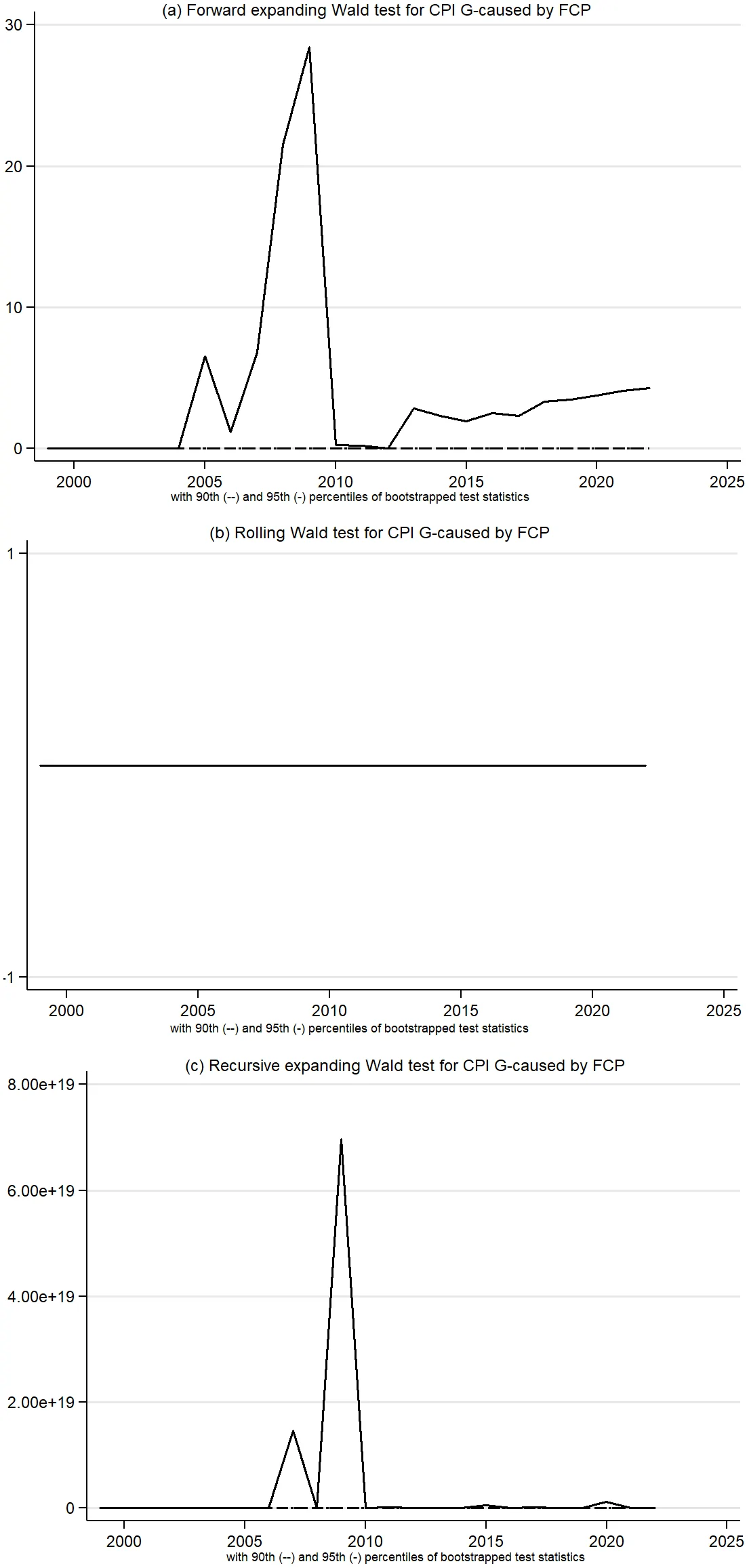
Food commodity prices Granger-cause the consumer price index. Robust estimation using VAR-based Granger causality test (Wald statistics) in the presence of instabilities.

**Fig 3:**
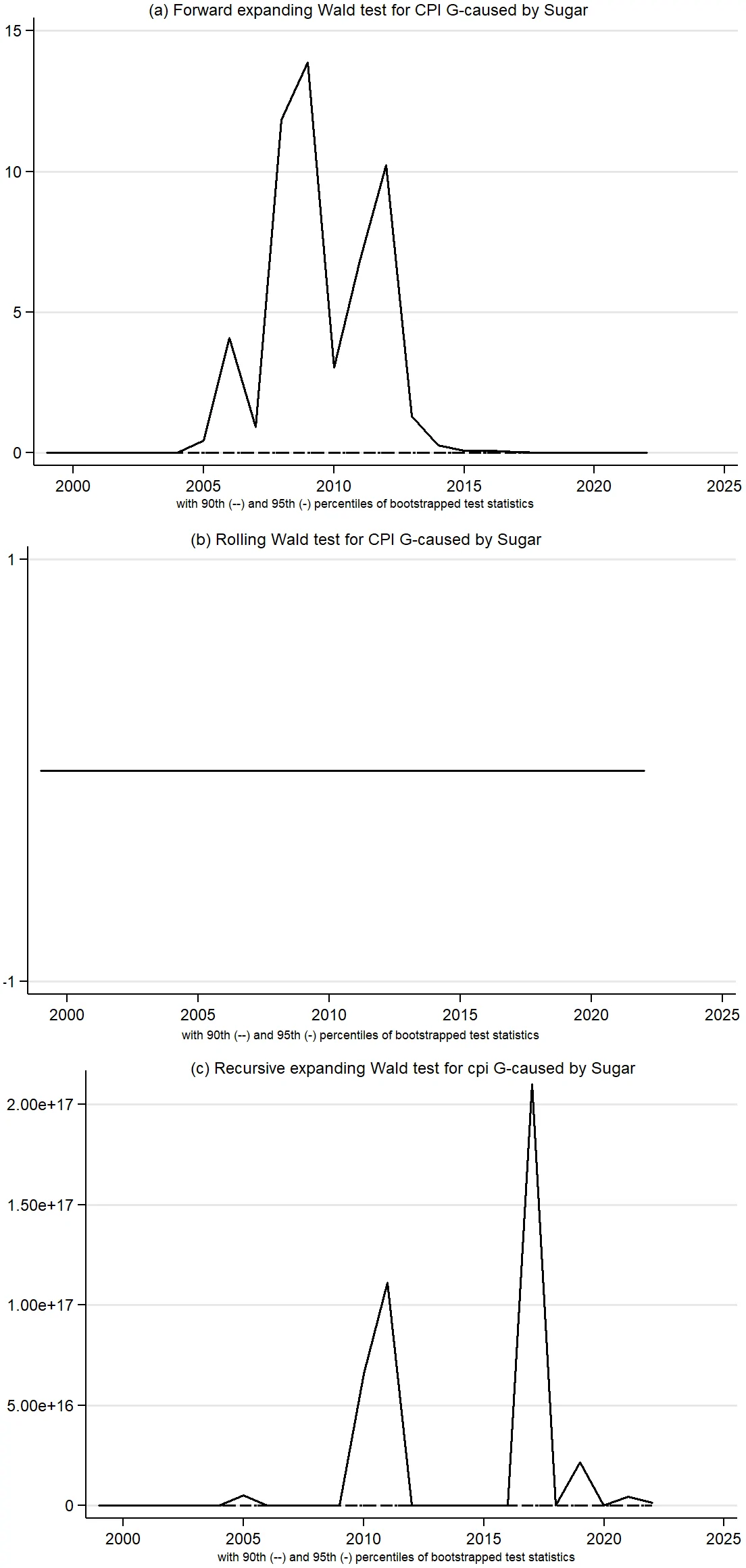
Sugar commodity prices Granger-cause the consumer price index. Robust estimation using VAR-based Granger causality test (Wald statistics) in the presence of instabilities.

In 2009, Somalia faced inflation mainly because of fluctuations in global food prices. Based on the FAO [57] report on global cereal markets, rising international food prices, particularly for cereals, led to persistently high domestic food costs in Somalia, worsening both inflation and food insecurity. The drought in 2011 was one of the most severe that the Horn of Africa has seen in six decades. The report of FAO [58] on the Horn of Africa crisis points out that rising global food prices, especially for cereals, played a major role in driving inflation in Somalia. The situation was made even worse by droughts that caused crop failures and disrupted trade routes, resulting in skyrocketing local cereal prices. These high prices dramatically lowered what households could afford, pushing the country deeper into famine. Alongside ongoing conflict and restricted access for humanitarian aid, the global surge in food costs exacerbated Somalia’s already severe food insecurity and inflation challenges. Somalia experienced yet another significant drought between 2016 and 2017, which devastated both crop production and livestock. This situation resulted in severe food shortages and rising prices. UN OCHA [59] reports that the ongoing conflict and road blockades have made it tough to get food into areas hit hard by drought. This disruption has led to higher operational costs and soaring prices for consumers. On top of that, crop failures and livestock losses due to the drought have made the food shortage even worse. Consequently, communities have had to rely more on costly imports, which have pushed basic food items out of reach for many people and have intensified inflation, particularly in remote regions. Between 2021 and 2022, Somalia faced its most severe drought in 40 years, compounded by worldwide issues in food supply. FAO-OCHA-UNICEF-WFP [60] highlights that rising global food prices, driven in part by the Ukraine conflict and disrupted supply chains, have significantly worsened inflation and food insecurity in Somalia. With local food and commodity prices surging, access to necessities like food and milk has sharply declined, especially for children. These price shocks, combined with failed rains and limited humanitarian funding, have created a “perfect storm” pushing Somalia toward famine, leaving millions unable to afford adequate nutrition and intensifying inflation across the country. COVID-19 also worsened Somalia’s food crisis by disrupting supply chains, weakening livelihoods, and adding to economic instability.

### 4.4 Kernel-based regularized least squares result

The KRLS results of the study are reported in Table 4. It presents the results of two models. The first model reports the impact of climate change, aggregate global food commodity prices, and political stability on inflation in Somalia, whereas the second model analyzes the impact of disaggregated global food commodity prices, such as sugar, cereals, meat, and oil prices. In model 1, it was found that rainfall, temperature, food commodity prices, political stability, and economic growth exert significant effects on inflation in Somalia. On average, an increase in rainfall tends to decrease inflation by about 0.18%. It also reduces inflation by about 1.35% and 0.61% in the 25^th^ and 50^th^ percentiles. But in the 75^th^ percentile, it raises inflation by 0.616%. Rainfall has a heterogeneous effect on inflation in Somalia. On the contrary, an average increase in temperature stimulates inflation by about 5.77%. Average temperature reduces inflation by about 9.66% in the 25^th^ percentile before it increases inflation by about 6.3% and 22% in the 50^th^ and 75^th^ percentiles, respectively. Similarly, temperature has heterogeneous marginal effects on inflation in Somalia. Furthermore, an average increase in global food commodity prices increases inflation by about 0.176%. In the 25^th^ percentile, it decreases inflation by about 0.23%; before it raises inflation by 0.22% and 0.60% in the 50^th^ and 75^th^ percentiles, respectively. This implies that a global food commodity price upsurge induces heterogeneous marginal effects on inflation in Somalia. Contrary to the heterogeneous marginal effects of rainfall, temperature, and food commodity prices, political stability has a homogeneous decreasing marginal effect on inflation in Somalia. A unit increase in political stability tends to decrease inflation by 0.15%, whereas it reduces inflation by about 0.43%, 0.12%, and 0.023% in the 25^th^, 50^th^, and 75^th^ percentiles, respectively. Finally, economic growth tends to raise inflation by about 0.64% for an average increase in output. Similarly, output growth stimulates inflation by increasing the cost of the basket of goods by 0.32%, 0.70%, and 95% in the 25^th^, 50^th^, and 75^th^ percentiles, respectively. Output growth has an increasing homogeneous marginal effect on inflation in Somalia.

**Table 4:** KRLS results.

Consumer price index	Model 1	Model 2
Rainfall		
Avg.	−.188***	.1631***
P25	−1.357***	−.297***
P50	−.398***	.239***
P75	.616***	.691***
t-statistics	(−16.111)	(16.851)
Temperature		
Avg.	5.777***	4.558***
P25	−9.668***	−4.728***
P50	6.371***	4.08***
P75	22.208***	18.841***
t-statistics	(69.243)	(50.676)
Global food commodity price		
Avg.	.176***	–––
P25	−.230***	
P50	.227***	
P75	.604***	
t-statistics	(63.984)	
Political stability		
Avg.	−.1511***	−.144***
P25	−.438***	−.361***
P50	−.122***	−.114*** -
P75	−.0023***	.035***
t-statistics	(−71.040)	(−150.161)
Real gross domestic product		
Avg.	.645***	.607***
P25	.322***	.339***
P50	.701***	.556***
P75	.953***	.892***
t-statistics	(275.950)	(304.775)
Sugar commodity price		
Avg.	–––	.019***
P25		−.135***
P50		.059***
P75		.1743***
t-statistics		(8.854)
Oil commodity price		
Avg.	–––	.009***
P25		−.109***
P50		.060***
P75		.118***
Std.		(4.853)
Cereals commodity price		
Avg.	–––	−.027*** -
P25		−.193***
P50		.013***
P75		.079***
Std.		(6.895)
Meat commodity price		
Avg.		.234*** -
P25	–––	.042***
P50		.272***
P75		.491***
Std.		(38.814)
***Model metrics***		
Obs	27	27
Lambda	.00152	.00152
Tolerance	.027	.027
Sigma	5.000	8.000
Eff. Df	26.39	26.65
R^2^	1.000	1.000
Looloss	2.507	3.482
***Robustness***		
Pr(skewness)	0.6480	0.0111
Pr(kurtosis)	0.2402	0.0096
Adj chi^2^(2)	1.73	10.57

Notes: (.) denotes the estimated standard error, *** represents statistical significance at *p-value <0.01*, P25, P50, and P75 represent the quantile (0.25, 0.50, and 0.75) specification to validate the unconditional distribution of estimated parameters. Pr(skewness) and Pr(kurtosis) represent skewness and kurtosis tests for normality.

To assess the robustness of the results on the global food commodity prices-inflation nexus in Somalia, we employed disaggregated variables of food commodity prices. The selection of these variables is based on data availability and the volume of these commodities that Somalia imports. The results are reported in Table 4 (Model 2). It was observed that an average increase in rainfall increases inflation by about 0.16%, even though it decreases inflation by 0.29% in the 25^th^ percentile. Rainfall exerts a positive effect on inflation in the 50^th^ and 75^th^ percentiles. Average temperature has produced similar results as in the first model. Temperature hampers macroeconomic stability by raising inflation in all percentiles except in the 25^th^ percentile. Similarly, political stability contributes to the reduction of inflation in all percentiles except in the 75^th^ percentile. Economic growth has a positive and increasing homogeneous marginal effect on inflation in Somalia. Sugar and oil prices contribute to the increase in inflation in Somalia by about 0.019% and 0.009%, respectively, for an average increase in these prices. Sugar and oil prices provide relief for consumers by decreasing inflation by about 0.13% and 0.10% in the 25^th^ percentile, even though they hamper the purchasing power of consumers by increasing inflation in the 50^th^ and 75^th^ percentiles. Notably, both induce heterogeneous increasing marginal effects on inflation in Somalia. These results contribute to the derivation of necessary policy implications for monetary authorities of the federal government of Somalia. Moreover, an average increase in cereal commodity prices increases inflation by about 0.027%. Similarly, cereals enhance the purchasing power of consumers by decreasing inflation by 0.19% in the 25^th^ percentile. But in the 50^th^ and 75^th^ percentiles, it increases inflation. Finally, an average increase in meat commodity prices exacerbates inflation by increasing the consumer price index by about 0.234. In a similar vein, meat prices hamper purchasing power by raising inflation in the 25^th^, 50^th^, and 75^th^ percentiles, which implies that it has increasing homogeneous marginal effects on inflation in Somalia.

To assess the adequacy of the estimated models, several diagnostics of the machine-learning algorithm were conducted, such as lambda, tolerance, goodness-of-fit, and log loss, as shown in Table 4 (Models 1 & 2). In Kernel Regularized Least Squares, λ (lambda) serves as a regularization parameter that penalizes excessively complicated or irregular functions within the reproducing kernel Hilbert space. This approach mitigates the risk of model overfitting and guarantees smoother, generalizable predictions. A small λ signifies that the model has significant flexibility to accommodate any small change—complex yet potentially overfitted. A large λ indicates that the model attains a smoother fit, disregarding tiny fluctuations—simpler although perhaps excessively rigid. KRLS selects λ, frequently by cross-validation, to achieve an optimal equilibrium between flexibility and generalization. Tolerance aims to achieve convergence by constraining the sensitivity of lambda through optimization. Goodness-of-fit quantifies the extent to which the explanatory factors account for the dependent variable. LOO loss is the aggregate of the squared leave-one-out errors. The estimated residuals of the data are normally and identically distributed in both models, as illustrated in Figs 4 and 5. Conversely, the CUSUM tests, illustrated in Figs 6 and 7, evaluated parameter stability. The results confirm that the residuals from the models are independent, and the CUSUM plots within the 95% confidence interval show that the model’s residuals stay stable over time without any major changes.

**Fig 4:**
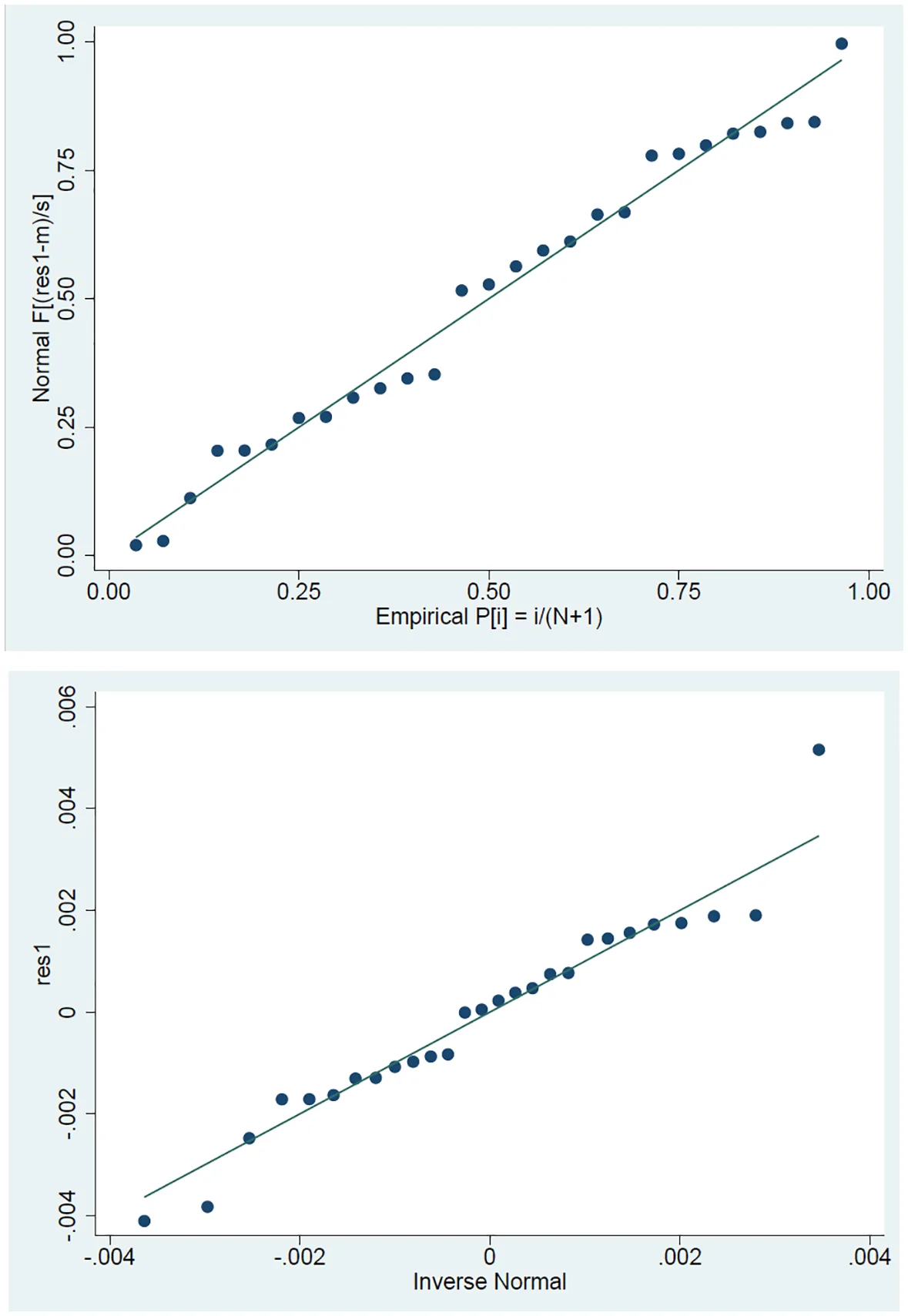
Normal distribution of the estimated residuals of Model 1.

**Fig 5:**
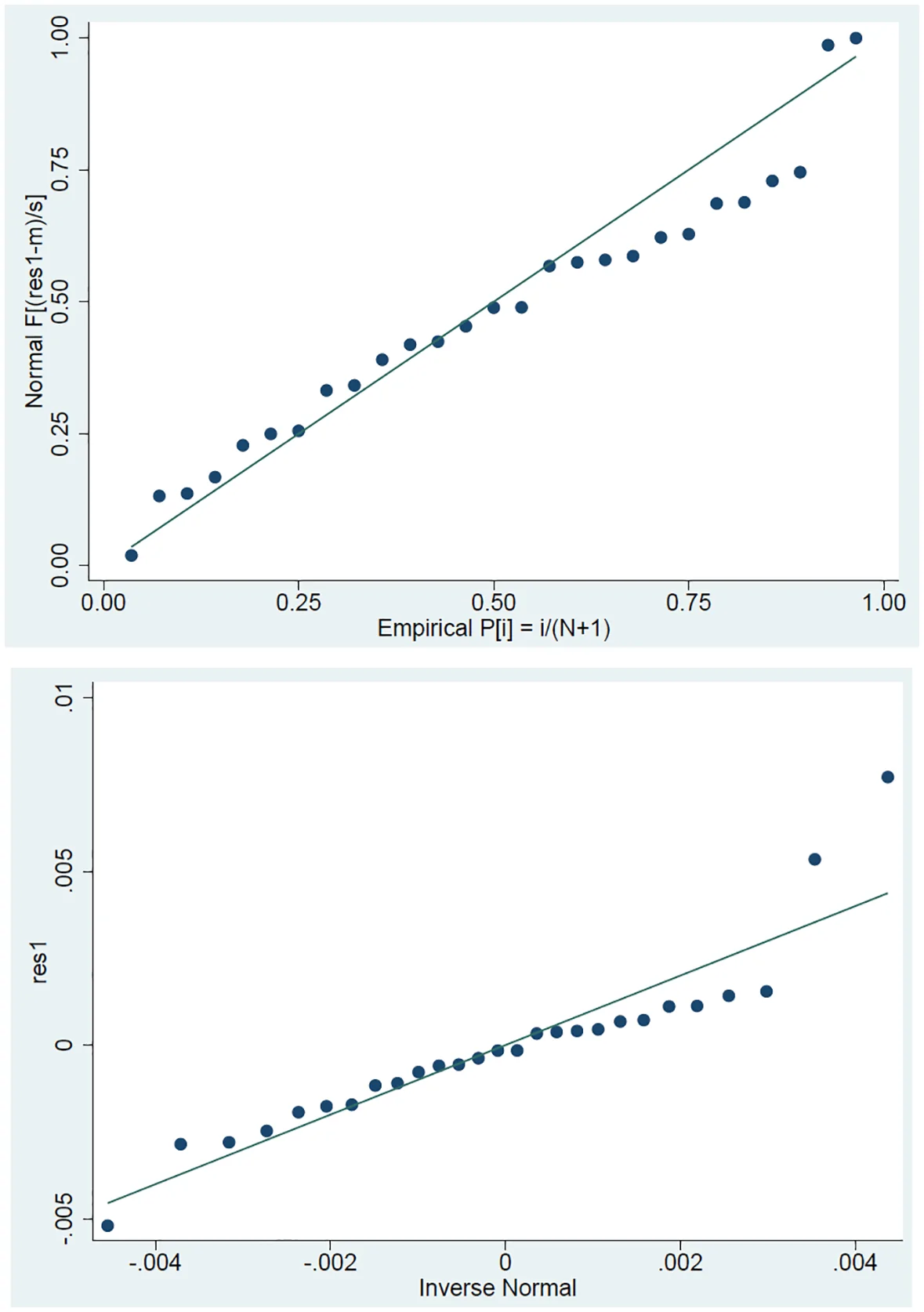
Normal distribution of the estimated residuals of Model 2.

**Fig 6:**
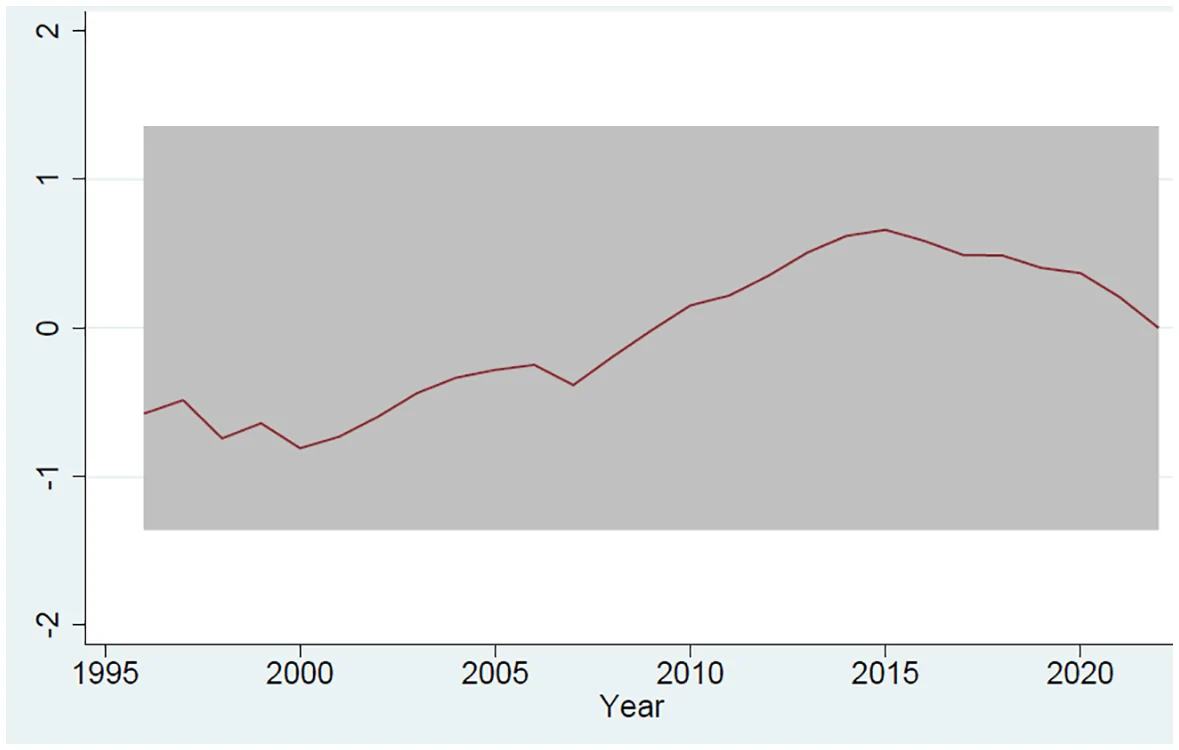
Parameter stability of the estimated residuals (Model 1).

**Fig 7:**
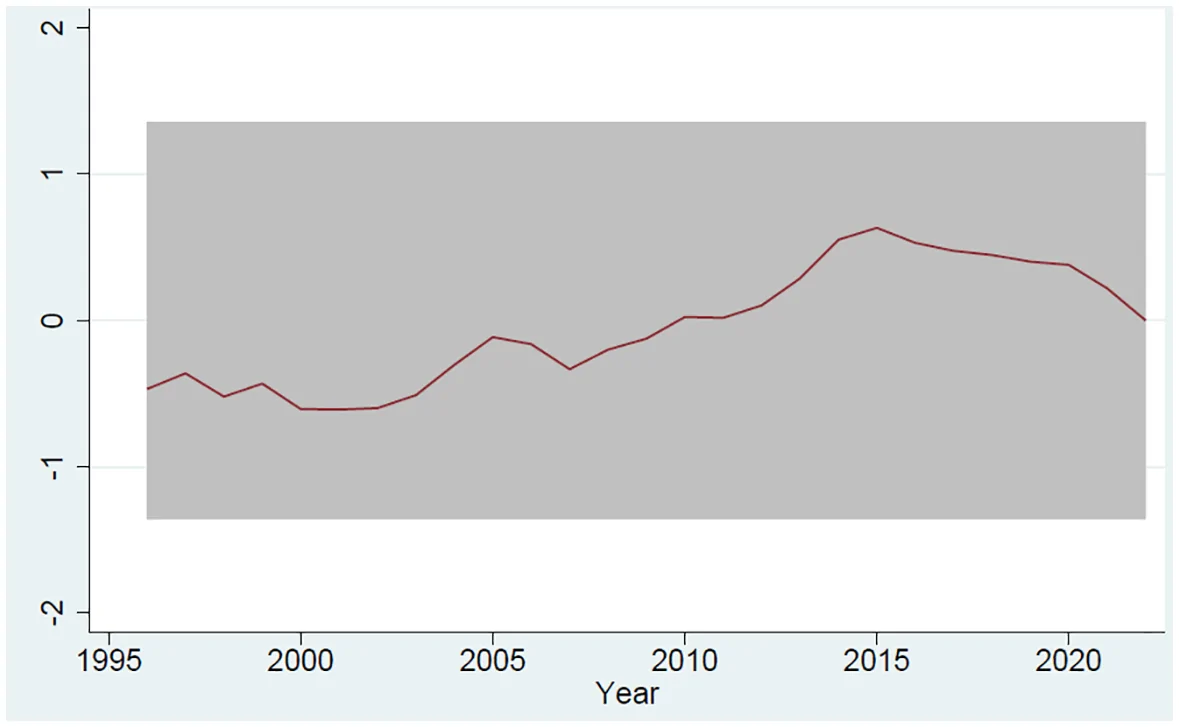
Parameter stability of the estimated residuals (Model 2).

### 4.5 ARDL F-bounds test

To assess the long-term cointegration among the series, we applied the bounds testing approach. The outcomes of the F-bounds cointegration test are presented in Table 5. According to the model’s findings, the calculated F-statistic is 4.882, which surpasses the upper bound critical value at the 1% significance level. This suggests the presence of a significant long-term cointegration relationship among the variables.

**Table 5:** ARDL bounds test.

F-statistic	4.882	K (5)
	Critical Value Bounds	
Significance	I (0) Bound	I (1) Bound
10%	2.08	3
5%	2.39	3.38
2.5%	2.7	3.73
1%	3.06	4.15

### 4.6 Long and short-run findings

Table 6 signifies the long-run and short-run relationship among variables. More precisely, the long-run results reveal that the food price index and gross domestic product have a positive and significant impact on Somalia’s inflation in the long run, while average temperature has a positive and insignificant impact. Conversely, average rainfall and political stability have a negative effect on inflation. Interpreting a 1% rise in the food price index and gross domestic product, inflation will increase by 0.515% and 0.844% in the long run, respectively. On the other hand, a 1% increase in average rainfall leads the inflation rate to decrease by about 0.885% in the long run. Finally, political stability improves purchasing power by decreasing the inflation rate by about 0.246% in the long run.

**Table 6:** ARDL long- and short-run results.

Variable	Coefficient (t-Statistic)
Long-run results	
lnAT	3.247 (0.616)
lnAR	−0.885* (−1.885)
lnFPI	0.515*** (3.723)
lnGDP	0.844*** (10.273)
PS	−0.246*** (−5.277)
Constant	−23.192 (−1.367)
Short-run results	
Δ (lnAT)	1.077 (0.371)
Δ (lnAR)	−0.364* (−1.955)
Δ (lnFPI)	0.114 (0.695)
Δ (lnGDP)	0.767*** (3.404)
Δ (PS)	−0.162*** (−4.667)
ECT (−1)	−1.023*** (−5.915)

Furthermore, the short-run results show that gross domestic product has a significant positive effect on inflation, while average temperature and food price index have a positive but insignificant impact on Somalia’s inflation in the short run. On the contrary, average rainfall and political stability significantly reduce inflation in the short run.

### 4.7 Diagnostic tests and model stability

As presented in Table 7, residual diagnostic tests were conducted to evaluate the model’s adequacy. The findings indicate that the model successfully passed all diagnostic checks, confirming that the residuals follow a normal distribution and exhibit no signs of serial correlation, heteroscedasticity, or incorrect functional form. Additionally, the model’s parameter stability was assessed using the Cumulative Sum (CUSUM) and Cumulative Sum of Squares (CUSUM SQ) tests, as illustrated in Fig 8. The resulting plots show that both CUSUM and CUSUM SQ remain within the critical limits at the 5% significance level, thereby confirming the stability of the model estimates.

**Table 7:** Diagnostic tests.

Test	Statistic
Reset test	3.132 [0.075]
Heteroskedasticity test	1.109 [0.351]
Adjusted R-squared	0.97
Auto correlation test	0.503 [0.418]
Normality	0.581 (0.747)

T-statistic values are in parentheses (…). P-values are in […].

**Fig 8:**
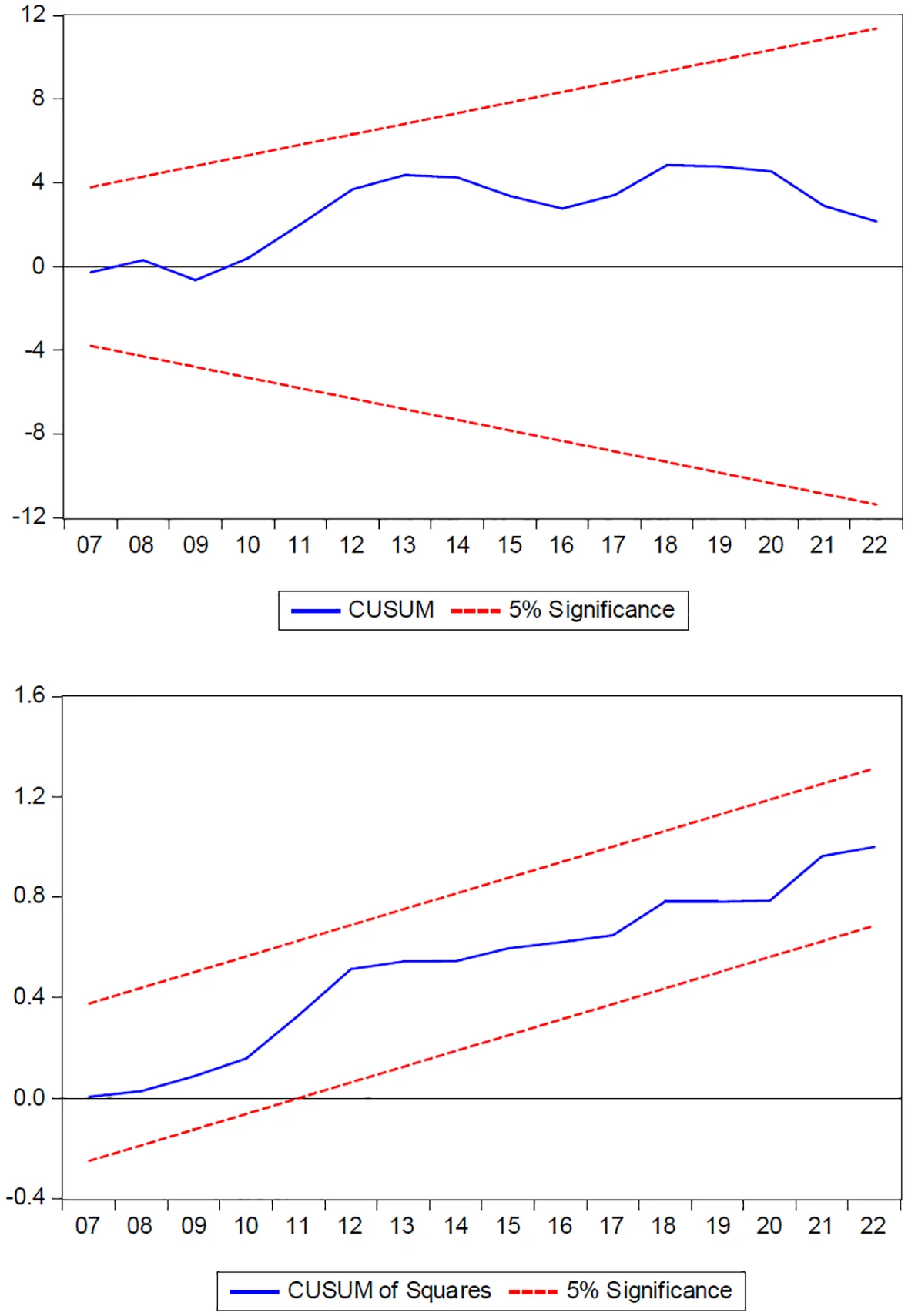
Model stability tests of CUSUM and CUSUM squares.

### 4.8 Discussion of the results

This study analyzed the macroeconomic determinants of inflation in Somalia with two distinct but complementary approaches: Kernel-based Regularized Least Squares (KRLS) and the Autoregressive Distributed Lag (ARDL) model. Each of them comes with different advantages regarding the intricacies of the Somali inflationary phenomenon. Together, they present a clear and complete picture of the country’s inflation dynamics. While KRLS excels in capturing nonlinear and heterogeneous marginal effects across different inflation levels, the ARDL offers insight into the short-run and long-run equilibrium relationships among the variables. Importantly, the findings from both models are largely consistent in direction and interpretation. This discussion combines both sets of results and analyzes their Somali economic implications.

Our finding that rainfall lessens inflation at the 25th and 50th percentiles while elevating it at the 75th percentile aligns closely with Somalia’s economic and climate patterns from 1996 to 2022. Somalia’s economy is primarily agrarian, with most of its population reliant on rainfall for farming and livestock production [20]. Due to food’s significant contribution to the consumer price index, precipitation patterns directly affect inflation. For instance, during low or moderate inflation, indicated by the 25th and 50th percentiles, sufficient rainfall generally boosts crop yields, promotes cattle grazing, and stabilizes food supply. This surplus contributes to lower food prices, alleviating import pressures, and ultimately mitigating inflation. This finding aligns with the finding of Odongo et al. [38], who found that average monthly rainfall significantly and negatively affects overall and food inflation. Nevertheless, during periods of elevated inflation—represented by the 75th percentile—the impact of rainfall is reversed. In Somalia, excessive or poorly timed precipitation frequently results in floods that devastate crops, impair infrastructure, and disrupt transportation and trade. When inflation is already high due to reasons like currency depreciation, conflicts, or global price shocks, these disruptions intensify supply shortages and drive prices upward. This result is consistent with the finding observed by Lefe et al. [61], who reported that precipitation has a positive impact on food security. Consequently, precipitation that is advantageous during stable conditions may become inflationary during periods of crisis. The heterogeneous effects estimated by KRLS elucidate this imbalance, demonstrating that the correlation between rainfall and inflation in Somalia is non-linear and contingent upon the overarching economic and meteorological context.

Temperature exhibits a strong positive association with inflation, predominantly increasing it. The inflationary impact of sustained elevated temperatures is less beneficial to agrarian output, livestock productivity, and water availability. Somali people, who already have to deal with irrigation challenges, face higher temperatures that worsen the situation by limiting food supply, thereby increasing the cost of food. Its effect is most obvious when inflation is extremely high, and the intersection between supply limitations and demand requirements is intensified. The economic significance of temperature lies in its role as a proxy for climate stress, which jeopardizes market stability and food security. The same result was revealed by Li et al. [39], who showed a clear positive relationship between rising temperatures and inflation, a finding that holds across various robustness checks. The analysis also reveals that the effect of temperature on inflation differs across countries, depending on their specific characteristics. One key mechanism identified is the increased energy demand caused by higher temperatures, which contributes to inflationary pressure. Erdogan et al. [41] also found that rising temperatures significantly drive food price inflation. On the contrary, Lefe et al. [61] observed that temperature is negatively associated with food security.

Furthermore, the study underscores the deep and long-lasting effects of global food commodity prices on local inflation. Somalia, which is a net food importer, remains vulnerable to shifts in the international market. Increases in global prices for core staples like sugar, cereals, and oil are quickly transmitted to the local market, because of heavy dependence on imports. The inflationary effect was severe during the worldwide food crisis of 2008, the disruptions caused by COVID-19, and recent geopolitical tensions. This highlights a structural vulnerability in Somalia’s economy, and the lack of mechanisms to protect against external shocks because of restrained domestic production, underdeveloped trade infrastructure, and fragile, easily disrupted supply chains. This finding aligns with the finding of Erdogan et al. [41], who discovered that oil prices, global food and agricultural prices considerably contribute to food price inflation. Asab [62], also showed that supply-side factors, particularly food and oil price shocks and supply chain disruptions, are the main drivers of inflation measured by both the Consumer Price Index (CPI) and the GDP deflator.

In the study, one of the most stabilizing forces identified was political stability. There is a clear correlation between political stability and inflation. Improvements in political stability foster an environment for business investment, which in turn enables the smooth working of markets and efficiency of economic policies. Economically, political stability serves as an anchor that minimizes the economy’s risk premium and volatility. It is a fundamental aspect of macroeconomic management as well as a governance goal. A similar finding was reported by Telatar et al. [48] indicates that reduced political instability leads to lower inflation, but this effect is observed only in developed countries and those with already low inflation levels. Similarly, the findings of Ghanayem et al. [46] highlighted that geopolitical risks stemming from the Russia-Ukraine conflict have further intensified inflationary pressures.

The study further highlights the significance of real GDP growth for inflation in the Somali context. While growth is a desirable development objective, findings indicate that increases in GDP are associated with rising inflation, indicating a demand-pull effect. This is the situation when rises in income and consumption are not matched by growth in the supply of goods and services. Given that Somalia’s productive base is quite limited and its supply chains are fragile, expansion in the economy can easily result in acute shortages alongside inflated prices. This becomes more acute in the case of sectors with the weakest domestic suppliers and imports are the primary source of supply. Hence, inflation results from a lack of control in an economy with unbalanced growth. Relatedly, Islam [63] found that economic growth, measured by gross domestic product (GDP) growth, leads to an increase in inflation.

Finally, more insights can be extracted from the disaggregated analysis of food commodities. Oil, meat, sugar, and other cereals each have a different level of contribution to inflation. The strongest effects were seen in meat and sugar, as they are most popular and among the top imports. The inflationary impact of these goods captures not just their significance in household expenditure but also their vulnerability to global market fluctuations. For example, prices of sugar increase during world supply disruptions, and meat prices are sensitive to the domestic pastoral situation and regional commerce patterns. A similar result was reported by Malaviarachchi and Korale-Gedara [64], who found that fluctuations in agricultural wages, fuel prices, money supply, and fertilizer costs are major factors contributing to food inflation in Sri Lanka. Irawati et al. [43] also revealed that rice, red chilies, and onions had a positive and significant influence on inflation in North Sumatera Province, whereas garlic showed no significant effect on inflation in the region.

## 5 Conclusion and policy implications

The aim of this study is to examine the impact of climate change, political stability, and global food commodity prices on inflation in Somalia using a mix of advanced econometric techniques. These include the Kernel-based Regularized Least Squares (KRLS), Autoregressive Distributed Lag (ARDL) models, and a time-varying Granger causality analysis, which was preceded by a supremum right-tail Augmented Dickey-Fuller (ADF) test to check for explosive behavior. These approaches were chosen to effectively capture both nonlinear effects and dynamic causal relationships in a setting characterized by structural breaks, shifts in regime, and high volatility. The findings indicate that inflation in Somalia is significantly affected by global food prices, climate variations, political stability, and economic growth. The analysis, using both KRLS and ARDL models, reveals that rainfall tends to lower inflation, while factors like rising temperatures, increasing global food prices, and economic growth contribute to higher inflation. Political stability consistently helps keep inflation in check across various models. Additionally, the explosive behavior test pinpointed unusual inflation episodes during global crises, specifically in the years 2009 and 2011. The time-varying Granger causality analysis further confirmed that global food prices, particularly for cereals and sugar, were significant predictors of inflation during crucial times, which aligns with Somalia’s reliance on imports.

Based on the findings, the study highlights several crucial policy directions to help manage inflation and stabilize prices in Somalia. First, there’s an urgent need to boost the resilience of the agricultural sector against climate variability. Adequate rainfall plays a significant role in keeping inflation in check by increasing food supply. However, both too much and too little rainfall can disrupt production and drive prices up. Therefore, the government should invest in irrigation systems, improve water management, and promote drought-resistant crops to ensure reliable food production and minimize vulnerability to climate-induced inflation. Second, rising temperatures also create inflationary pressures by decreasing agricultural output and challenging water and livestock systems. Somalia needs to implement comprehensive climate adaptation strategies, such as promoting heat-tolerant crops, expanding irrigation access, and training farmers to cope with climate stress. Third, reducing reliance on imported food is crucial to shield the economy from global price shocks. The study reveals a strong link between increases in international food prices, particularly for sugar, meat, cereals, and oil, and domestic inflation. Thus, Somalia should work on bolstering local food production, creating strategic food reserves, securing stable trade agreements, and setting up a national food price monitoring system to enhance resilience against external shocks.

Fourth, political stability is also a key factor in maintaining price stability. Political stability helps reduce inflation by improving market efficiency, boosting investor confidence, and ensuring consistent policies. Consequently, reinforcing governance, fighting corruption, and fostering peace are not just political objectives but also vital economic strategies for controlling inflation. Fifth, while economic growth is crucial, it is important to note that growth without adequate domestic capacity can lead to inflation. Somalia needs to invest in infrastructure, support rural producers, and develop food processing and manufacturing industries to ensure that growth benefits everyone and is supply-driven, helping to avoid inflationary pressures as incomes rise. Certain commodities like meat and sugar have a notable impact on inflation and require targeted policy interventions. Temporary subsidies, support for local production, and effective price monitoring should be prioritized. Establishing a national food price observatory can help track risks associated with essential goods and inform policy decisions more effectively. Lastly, tackling inflation in Somalia requires better coordination among institutions. Given the complex nature of the factors contributing to inflation, ranging from climate issues to governance and trade, it’s crucial to set up a national inflation response committee or inter-ministerial task force. This team should coordinate efforts across key sectors, including agriculture, finance, trade, planning, and environmental protection, into a cohesive and integrated strategy. Only through unified, cross-sector coordination can Somalia effectively address the underlying causes of inflation and achieve long-term price stability.

Our study is not immune from limitations. One of the limitations of this study is its application of a small sample size (1996–2022); hence, we recommend that future studies utilize a larger sample size

and incorporate other significant macroeconomic variables, such as exchange rates. Moreover, we also recommend using simultaneous models to examine the causal relationships among climate change, the international economy, the political economy, and inflation.
